# Who is a good psychiatrist?—A collective view

**DOI:** 10.4103/0019-5545.55949

**Published:** 2005

**Authors:** Nirmala Srinivasan

‘Who is a good psychiatrist?’ is the irresistible voice of concern for most family members of persons suffering from chronic mental disorders. When they confront each other in the waiting room of the doctor's clinic, notes are exchanged in hushed tones almost on the same wavelength about the patient and the doctor. ‘Is he good?’, ‘Does he talk to the patient nicely or is he rude?’, ‘Does he receive your phone calls?’ Even the first visit is chosen with a lot of caution and care, almost like looking for a marital alliance for a son or daughter. Being a caregiver myself, I am aware of the elaborate enquiries that many families make in choosing a therapist, especially in the initial stages. Discreet enquiries are made of those friends and relatives who will keep the oath of secrecy. Compatibility with the patient, distance to travel for an appointment, consultation fees, professional reputation are looked into with abundant precaution before zooming in on a particular professional. It is not necessary that the initial choice is the final one. Contrary to professional advice, parallel consultations and specialist hopping go on—a privilege enjoyed by clients in metropolitan towns.

Changing of psychiatrists is an issue by itself. Most families go by the first choice, especially if there is some remarkable improvement, even in a chronic case. One mother told me that she went to another doctor after 12 years because ‘the doctor was looking at it as an old case of schizophrenia even though the symptoms have changed. He does not listen. So I decided to go to another one and I was right’. Probably, changing the therapist is more difficult than making the initial choice. Families do so under severe pressure either from their patients or due to other circumstances. Though rare, it is a phenomenon to reckon with.

An interesting indicator of the benchmark of a good psychiatrist is FAQs of family members. According to Professor Reuven Bar-On, psychiatrists ought to rank first on the list of professionals with a high EQ. (Professor Reuven Bar-On of the University of Haifa in Israel, had prepared an elaborate list of professions ranked according to the expected levels of EQ.) Strong coincidence between his professional worldview and that of family caregivers and consumers (or patients) cannot be ruled out as seen from the views expressed by caregivers, which are discussed below.

This topic, which continues to be of perennial interest to patients and their family members, was discussed in one of the monthly meetings of AMEND, a self-help family support group in Bangalore that was started by my family in 1992. Though informal in nature, the group talk unravelled many interesting expectations of family members about the profile of a good psychiatrist. The main objective was to assess and compare diverse views of family members on the qualities that made up the profile of a good psychiatrist. For the discussion there were 35 persons representing about 22 families, mostly parents. I anchored the workshop using the method of freewheeling discussions to elicit the best response from each individual or family, based on their experiences. The responses were grouped into four categories: (i) general demeanour, (ii) skill sets, (iii) personality traits, and (iv) subject knowledge.

## GENERAL DEMEANOUR

- Is possessed with the zeal and stamina to be alert during consultations
- Well-dressed (neither too formal nor too casual; not in jeans, smoking, etc.)
- Is warm and does make the patient comfortable (there may be exceptions, especially if the two do not belong to the same sex). Family members and a few patients who participated expressed a preference for a doctor of the same sex as that of the patient.
- Is pleasant in an emergency and also easily accessible, in the hospital/clinic/residence (mobile or even a phone call), particularly in emergencies.

## SKILL SETS (FOR CONSULTATION SESSIONS)

- Listens with a keen ear to the symptoms
- Communicates with ease
- Uses body language in a positive way—smiles or greets, with a handshake or even an encouraging pat
- Bonds with the client's family by inspiring confidence and trust—‘Do not worry. I am there’ assurance
- Is polite but firm
- Demonstrates leadership skills when the client's family is confused and lost
- Has the ability to be a team player with the patient, family and other professionals
- Is open-minded
- Respects the caregiver's and patient's right to information about treatment

## PERSONALITY TRAITS

- Remains calm, unruffled, especially in a crisis
- Exudes an air of quiet competence
- Is mature and behaves predictably
- Is not rude or abrasive; gets his/her job done in an assertive manner without being angry with the family members
- Is highly intuitive and alert in making analytical connections
- Is compassionate and caring
- Is positive and optimistic
- Feels and demonstrates genuine respect for the patient's family as partners
- Is open-minded and willing to clarify doubts and offer logical explanations about treatment, choice of medication, etc.

## PROFESSIONAL EXPERTISE

- Is good in the practical application of theory
- Keeps abreast of the latest developments in the field
- Keeps families informed about recently introduced medicines
- Commands respect in the profession
- Participates in advocacy aspects by initiating information to families.

The findings indicate that there is a strong tendency to idealize the role of a psychiatrist, probably because he or she can make the difference between a happy and an unhappy family. Also, perhaps, for family members, the profession is believed to reflect the fine balance between emotions and rationality.

My voice is our collective voice.

